# A MALDI-TOF MS-based multiple detection panel of drug resistance-associated multiple single-nucleotide polymorphisms in *Candida tropicalis*

**DOI:** 10.1128/spectrum.00764-24

**Published:** 2024-12-06

**Authors:** Feifei Wan, Min Zhang, Jian Guo, Huiping Lin, Xiaoguang Zhou, Lixin Wang, Wenjuan Wu

**Affiliations:** 1 Department of Laboratory Medicine, Shanghai East Hospital, School of Medicine, Tongji University, Shanghai, China; 2 Intelligene Biosystems (Qingdao) Co., Ltd, Qingdao, China; University of Lagos, Lagos, Nigeria

**Keywords:** *Candida tropicalis*, matrix-assisted laser desorption/ionization-time of flight mass spectrometry, single-nucleotide polymorphism

## Abstract

*Candida tropicalis* is one of the main causes of invasive candidiasis. Rapid identification of antifungal resistance is crucial for selection of an appropriate antifungal to improve patient outcomes. Mutations at specific loci are strongly correlated with resistance to antifungal agents. In this study, we developed a multi-single-nucleotide polymorphism (SNP) panel to accurately identify 36 mutation sites across seven genes of *C. tropicalis* that are associated with resistance to azoles and/or echinocandins. Ten isolates were selected to test repeatability, and another 20 isolates of *C. tropicalis* were selected to validate consistency. Intra-assay and inter-assay repeatability of the panel was 100%, with the loci accuracy being 99.44% (716 of 720). Furthermore, 109 isolates were examined for clinical research, and the most commonly detected mutations were G751A and A866T of *UPC2*, A491T of *TAC1*, and A395T and C461T of *ERG11*. The G751A and A866T mutations of *UPC2* as well as the A395T and C461T mutations of *ERG11* co-existed. The SNP panel enables identification of specific mutations at critical sites of drug-resistant strains to facilitate the rapid selection of appropriate antifungal agents and efficient monitoring of the regional epidemiological trends of resistance of *C. tropicalis*.

**IMPORTANCE:**

*C. tropicalis* infections pose a growing global public health challenge, with mortality rates approaching 40%. *C. tropicalis* is one of the top four *Candida* spp. responsible for candidiasis, particularly in the Asia-Pacific region and Latin America, notably affecting patients with neutropenia and malignancies. The azole resistance rate of *C. tropicalis* ranges from 0% to 30%. Between 2009 and 2018, the China Hospital Invasive Fungal Surveillance Network reported an increase in fluconazole and voriconazole resistance from 5.7% to ~30%. Although resistance to echinocandins and amphotericin B remains low, multi-resistance to echinocandins and azoles has been observed. Current methods for detecting drug resistance are limited by the long turnaround time of antifungal susceptibility testing, low throughput of Sanger sequence to target resistance mutations, complex data analysis, and high costs of second-generation sequencing. We developed and validated a rapid, high-throughput, and cost-effective panel to detect and monitor drug-resistance mutations of *C. tropicalis*.

## INTRODUCTION

Invasive candidiasis (IC) refers to systemic fungal infections caused by *Candida* spp., including candidemia and deep-seated infection (1). Mortality of IC can be as high as 40%, especially in immunocompromised, profound neutropenic, and intensive care unit (ICU) patients. Approximately 50% of candidaemia cases occur in the ICU (1, 2). While more than 15 distinct *Candida* spp. can cause human infections, the 6 most clinically relevant are *Candida albicans*, *Candida parapsilosis*, *Pichia kudriavzevii*, *Nakaseomyces glabratus*, *Candida tropicalis*, and *Candida auris* (3). *C. tropicalis* is associated with high morbidity and mortality due to the formation of strong biofilms, high virulence, hospital horizontal transmission, and antifungal resistance (4, 5). Recent evidence indicates an azole resistance rate of 20%–50% for *C. tropicalis* in the Asia-Pacific region, India, Turkey, and Algeria (6–9). The emergence of fluconazole resistance has led to increased echinocandin exposure, with *C. tropicalis* isolates resistant to both azoles and echinocandins being reported in the USA (10, 11), India (7), Taiwan (12), and mainland China (13), although the prevalence remains low (<2%).

Azoles and echinocandins are the first-line therapeutic agents for IC. Azoles targeting 14-alpha-demethylase (Erg11) inhibit the ergosterol biosynthesis pathway in fungal cell membranes, whereas echinocandins bind to the Fks p-subunit of the β-(1,3)-D-glucan synthase complex, thereby blocking synthesis of β-(1,3)-D-glucan (14, 15). Azole resistance can develop through multiple mechanisms, including substitutions in Erg11, which reduce the drug-binding affinity between ergosterol and azoles (16, 17). Additionally, overexpression of Erg11, driven by gain-of-function mutations to the transcriptional activator *UPC2*, can lead to overexpression of drug targets. Azole resistance is also associated with upregulation of drug efflux pumps. Mutations to *TAC1* or *MRR1* lead to the upregulation of adenosine triphosphate-binding cassette transporter *CDR1* and the major facilitator superfamily transporter *MDR1*. Resistance to echinocandins primarily involves mutations to *FKS1* (16).

The broth microdilution method is recommended as the reference method for antifungal susceptibility testing to determine the minimal inhibitory concentration (MIC) by the Clinical and Laboratory Standards Institute and the European Committee on Antimicrobial Susceptibility Testing (18, 19). However, this method is limited by a long turnaround time, tedious procedures, and subjective interpretation of the results. Recent advancements in research on drug resistance mechanisms of *C. tropicalis* have led to the development of molecular biology methods to detect resistance-related mutations, aiming to clarify antifungal resistance. Sanger sequencing is regarded as the gold standard. However, its single-target nature makes it relatively time consuming and costly. Considering the emergence of azole resistance of *C. tropicalis* and increased exposure to echinocandins, developing alternative molecular methods is essential for rapid, reliable, accurate, and cost-effective monitoring of antifungal resistance to optimize treatment outcomes.

Matrix-assisted laser desorption ionization time-of-flight mass spectrometry (MALDI-TOF MS) was first used for detection of single-nucleotide polymorphisms (SNPs) in human genomic DNA in 1998 (20) and more recently has been applied for genotyping of SNPs of human herpesviruses, human papillomavirus, methicillin-resistant *Staphylococcus aureus*, and *Mycoplasma pneumoniae* (21–24). The advantages of MALDI-TOF MS as an alternative molecular biology method for genotyping SNPs include high throughput, real-time analysis, accuracy, precision, and cost-effectiveness compared to other methods.

Here, we report the development of a multi-SNP detection panel based on MALDI-TOF MS to identify the drug-resistant phenotypes and facilitate epidemiological studies of the SNPs of *C. tropicalis* genes associated with antifungal resistance.

## RESULTS

### Repeatability evaluation

The repeatability of the panel was assessed using 10 *C*. *tropicalis* isolates. Intra-assay repeatability was evaluated using three technical replicates per isolate, and inter-assay repeatability was assessed across two independent experiments. All replicates across both intra-assay and inter-assay conditions demonstrated complete concordance at all loci. As no discrepancies were observed, the panel exhibited 100% repeatability.

Overall, mutations were detected at nine distinct loci of four genes, including T1949C of *FKS1*; A395T, T433C, and C461T of *ERG11*; G751A, A866T, and C1178T of *UPC2*; and A491T of *TAC1*. Among the tested isolates, four (ECIFIG685, ECIFIG687, ECIFIG1112, and ECIFIG1831) were resistant to both fluconazole and voriconazole, involving SNPs of *ERG11* (A395T and C461T), *UPC2* (G751A and A866T), and *TAC1* (A491T). One additional isolate, resistant to fluconazole and intermediate to voriconazole, carried mutations to *UPC2* (G751A, A866T, and C1178T). An isolate, susceptible-dose dependent to fluconazole and intermediate to voriconazole, harbored the mutation T433C of *ERG3*. In contrast, isolates susceptible to both fluconazole and voriconazole (e.g., ECIFIG31, ECIFIG343, and ECIFIG1642) had fewer or no significant resistance-associated SNPs in *UPC2* (G751A and A866T) or *TAC1* (A491T). Notably, isolates ECIFIG1642 and ECIFIG21032, which were resistant to anidulafungin, lack resistance-associated SNPs of *FKS1*. Other isolates (except strain ECIFIG1112), which were susceptible to all three echinocandins, did not have resistance-associated SNPs of *FKS1* (Table 1).

**TABLE 1:** SNPs of 10 *C*. *tropicalis* isolates identified by MALDI-TOF MS and selected to evaluate repeatability^*a*^

Strain	Year of isolation	FKS1	ERG11			UPC2				TAC1	Susceptibility to echinocandins and azoles				
		1949 T→C	395 A→T	433 T→C	461 C→T	751 G→A	866 A→T	1178 C→T	1712 T→A	491 A→T	ANI	CAS	MICA	FLU	VOR
ECIFIG31	2017	T	A	T	C	G, A	A, T	C	T	T	S	S	S	S	S
ECIFIG343	2018	T	A	T	C	G, A	A, T	C	T	A	S	S	S	S	S
ECIFIG685	2018	T	A, T	T	C, T	G	T	C	T	T	S	S	S	R	R
ECIFIG687	2018	T	A, T	T	C, T	A	T	C	T	T	S	S	S	R	R
ECIFIG1112	2019	C	A, T	T	C, T	A	T	C	A	A, T	S	S	S	R	R
ECIFIG1448	2019	T	A	T	C	G	A	C	T	A	S	S	S	S	S
ECIFIG1642	2020	T	A	T	C	G	A	C	T	A, T	R	S	S	S	S
ECIFIG1831	2020	T	A, T	T	C, T	A	T	C	T	A, T	S	S	S	R	R
ECIFIG21032	2021	T	A	T	C	G, A	T	T	T	A	R	I	R	R	I
ECIFIG21340	2021	T	A	C	C	G	A	C	T	A	S	S	S	SDD	I

^*a*^ Abbreviations: ANI, anidulafungin; CAS, caspofungin; FLU, fluconazole; I, intermediate; MICA, micafungin; R, resistant; S, susceptible; SDD, susceptible-dose dependent; VOR, voriconazole.

### Coherence assessment

To evaluate the accuracy of the multiplex SNP detection panel for *C. tropicalis*, 20 *C*. *tropicalis* isolates were tested across 36 SNP loci, resulting in a total of 720 SNP evaluations. Strains ECIFIG159 and ECIFIG385 demonstrated discordance at a single SNP locus (*ERG11*-395 and *FKS1*-1958, respectively), while strain ECIFIG455 exhibited discordance at two loci (*UPC2*-751 and *UPC2*-787). The remaining 716 loci were found to be concordant with the reference method. The system demonstrated an overall accuracy of 99.44% (716 of 720) (Table 2).

**TABLE 2:** SNPs of 20 *C*. *tropicalis* isolates identified by MALDI-TOF MS and selected for coherence assessment

Strain	Year of isolation	FKS1		ERG11				UPC2			TAC1	MDR1	MRR1
		1958 T→C	1960 TC→CC	395^*a*^ A→T	433 T→C	461^*a*^ C→T	1286 C→T	751 G→A	787 G→A	866 A→T	491 A→T	227 T→C	1939 G→T
ECIFIG27	2017	T	TC	A	T	C	C	G	G	A	A,T	T	G
ECIFIG113	2017	T	TC	A	T	C	C	G	G	A	A,T	T	G
ECIFIG158	2017	T	TC	T	T	C,T	C	G,A	G	A,T	A,T	T	G
ECIFIG159	2017	T	TC	A (A,T)	T	C,T	C	G,A	G	A,T	A,T	T	G
ECIFIG189	2017	T	TC	A	T	C	C	G	G	A	A,T	T	G
ECIFIG196	2017	T	TC	A	T	C	C	A	G	T	A	T	G
ECIFIG214	2017	T	TC	A	T	C	C	G	A	A	A,T	T	G,T
ECIFIG260	2018	T	TC	A	T	C	C,T	A	G	T	A,T	T	G
ECIFIG354	2018	T	TC	A	T	C	C	A	G	T	A	T	G
ECIFIG358	2018	T	TC	A	T,C	C	C	G,A	G	A,T	A	T	G
ECIFIG385	2018	T,G (T)	TC	A	T	C	C	G	G	A	A,T	T	G
ECIFIG455	2018	T	TC	A	T	C	C	G (G,A)	G,A (G)	A,T	A	T	G
ECIFIG760	2018	T	TC	A	T	C	C	G,A	G,A	A,T	A,T	T	G
ECIFIG818	2018	T	TC	A	C	C	C	G	G	A	A	T	G
ECIFIG833	2018	T	TC	A	T	C	C	G	G	A	A,T	T	G
ECIFIG844	2018	T	TC	A	T	C	C	G	G	A	A,T	T,C	G
ECIFIG885	2019	T	TC	T	T	T	C	G,A	G	A,T	A,T	T	G
ECIFIG889	2019	T	TC	T	T	T	C	G,A	G	A,T	A,T	T	G
ECIFIG909	2019	T	TC	T	T	T	C	G,A	G	A,T	A,T	T	G
ECIFIG1521	2020	T	CC	T	T	T	C	G,A	G	A,T	A,T	T	G

^*a*^ SNPs associated with antifungal resistance (gene editing level). Mutations detected by MALDI-TOF MS are underscored. When the results detected by MALDI-TOF MS were inconsistent with the Sanger sequence, the latter results are indicated in parentheses.

All isolates had at least one mutation. Among the 36 loci examined, mutations were detected at 12 loci across six genes (Table 2). Due to the diploid nature of *C. tropicalis*, both homozygous and heterozygous mutations were detected.

### Clinical performance evaluation

**Fig 1:**
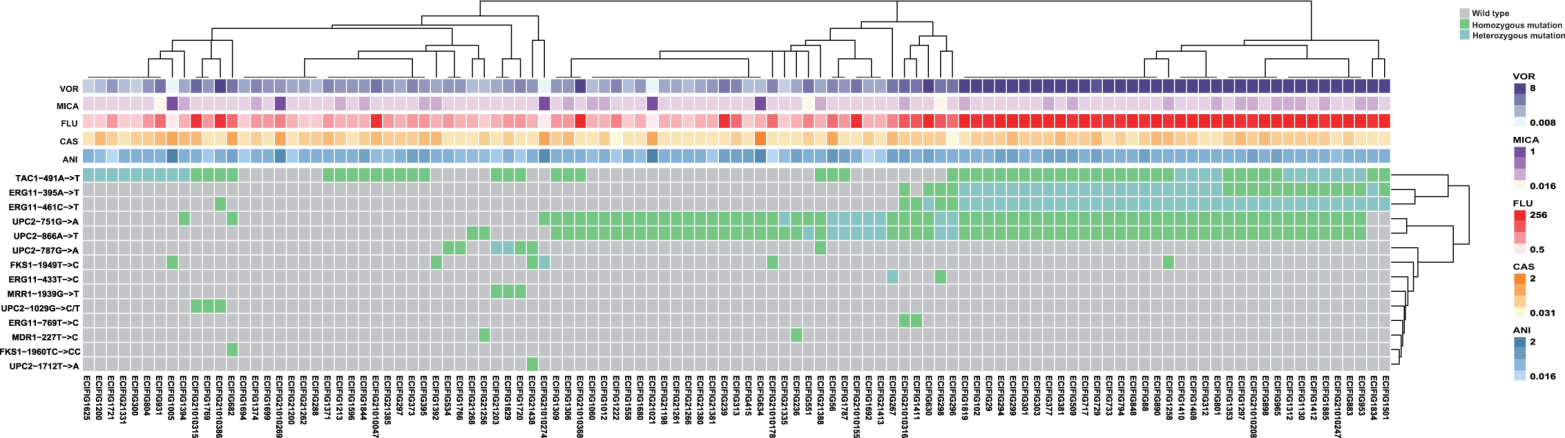
Cluster heat map of mutations of 109 *C*. *tropicalis* isolates. The heat map was generated based on the mutations at 14 loci of 109 *C*. *tropicalis* isolates. Gray indicates wild type; green indicates homozygous mutations; and blue indicates heterozygous mutations. The horizontal coordinates of the heat map represent the isolates, and the vertical coordinates represent the gene loci. The color annotation bar at the top demonstrates the antifungal agent susceptibility of each isolate of *C. tropicalis*. The MICs are listed in in Table S1.

Given the high consistency rate and reliability, this panel was further used to identify mutations of 109 *C*. *tropicalis* isolates. In total, 14 distinct loci with mutations were identified, which were mainly concentrated at loci *UPC2*-751 (65.14%, 71 of 109), *UPC2*-866 (64.22%, 70 of 109), *TAC1*-491 (62.39%, 68 of 109), *ERG11*-461 (38.53%, 42 of 109), and *ERG11*-395 (36.70%, 40 of 109). Additionally, co-existence of mutations at the loci *UPC2*-751 and *UPC2*-866, as well as *ERG11*-395 and *ERG11*-461, was frequent. Mutations at *ERG11*-395 and *ERG11*-461 were exclusive to azole-resistant *C. tropicalis*, whereas mutations at *UPC2*-751, *UPC2*-866, and *TAC1*-491 occurred in isolates resistant and not resistant to azoles. Among isolates with a mutation at the *ERG11*-461 locus, 95.24% also had a mutation at the *ERG11*-395 locus; 90.48% had a mutation at the *TAC1*-491 locus; and 92.86% had mutations at the *UPC2*-751 and *UPC2*-866 loci. Among isolates with a mutation at the *UPC2*-751 locus, 95.77% also had a mutation at the *UPC2*-866 locus; 60.56% had a mutation at the *TAC1*-491 locus; 53.52% had a mutation at the *ERG11*-395 locus; and 54.92% had a mutation at the *ERG11*-461 locus (Fig. 1).

The mutations at the loci *ERG11*-395, *ERG11*-461, and *ERG11*-769 were related to azole resistance, as confirmed by gene editing. In this study, all isolates with mutations at these loci exhibited azole resistance. For isolates with mutations at both the *ERG11*-395 and *ERG11*-461 loci, 90% (36 of 40) were highly resistant to fluconazole (MIC ≥128 g/L) and voriconazole (MIC ≥8 g/L). However, not all azole-resistant isolates carried these three mutations, as some carried mutations at other loci associated with azole resistance, such as *UPC2*-1029 (2.75%, 3 of 109), *UPC2*-751 (65.14%, 71 of 109), *TAC1*-491 (62.39%, 68 of 109), *ERG11*-433 (1.83%, 2 of 109), and *UPC2*-866 (64.22%, 70 of 109). Additionally, isolates with SNPs at the *UPC2*-1029 and *ERG11*-433 loci were not susceptible to fluconazole and voriconazole. However, these SNPs were absent in other isolates susceptible to fluconazole and voriconazole. Among the strains with mutations at both the *UPC2*-751 and *UPC2*-866 loci, 70.58% (48 of 68) were resistant to fluconazole and voriconazole. Notably, ECIFIG21010269 did not have any of the mutations and was sensitive to voriconazole but resistant to fluconazole (MIC = 8 g/L).

The SNPs at loci 1949 (5.50%, 6 of 109) and 1960 (0.91%, 1 of 109) of *FKS1* were associated with resistance to echinocandins. The SNP at locus 1949 of *FKS1* was identified in two isolates that were resistance to anidulafungin and micafungin, while the remaining four isolates were sensitive to echinocandins. The SNP at locus 1960 of *FKS1* was identified in one isolate, which exhibited intermediate resistance to caspofungi. Additionally, four isolates with mutations were not susceptible to echinocandins.

## DISCUSSION

*C. tropicalis* infections and the emergence of drug resistance present a significant threat to human health. There is an urgent need to develop rapid and accurate methods to detect antifungal agent sensitivity. Currently, microbroth dilution is recommended as the reference method forantifungal susceptibility testing, but this method requires a long turnaround time, making it impractical for situations that require quick results (25). Current molecular biology detection methods to detect drug resistance include polymerase chain reaction (PCR), PCR-restriction fragment length polymorphism analysis, PCR-single-strand conformation polymorphism analysis, TaqMan probe technology, gene chip technology, and next-generation sequencing (26–29). However, PCR detection sensitivity for SNP depends on the equipment, while next-generation sequencing is time consuming, complex, and requires expensive equipment. The resistance mechanisms of SNPs at specific loci are often multi-factorial, involving multiple SNP loci and different regulatory pathways (30). This complexity means that a single biomarker may not be sufficient to fully capture the drug resistance profile. Therefore, a combination of potential biomarkers is recommended to determine the antifungal susceptibility of *C. tropicalis* (31, 32). Multiplex PCR with single base pair extension is a useful technique for detection of more than 10 SNP sites and can be adopted for genotyping and identification of multiple microorganisms (33).

The developed panel based on MALDI-TOF MS combined with multiplex PCR and single base pair extension can accurately identify SNPs of *C. tropicalis* associated with resistance to azoles and echinocandins within 6–8 hours. The panel includes 36 SNP sites, including 9 related to azole resistance, as confirmed by gene editing. The accuracy of the panel was 99.44%, with intra-assay and inter-assay repeatability achieving 100%, demonstrating that the panel has potential for clinical detection of drug-resistant gene mutations in *C. tropicalis*.

The mutations of all 109 *C*. *tropicalis* isolates collected from eastern China were mainly concentrated at five sites: *UPC2*-751, *UPC2*-866, *TAC1*-491, *ERG11*-395, and *ERG11*-461. The co-occurrence of mutations at *UPC2*-751 and *UPC2*-866, as well as *ERG11*-395 and *ERG11*-461, was remarkably high. The mutations at the *ERG11*-395 and *ERG11*-461 loci were exclusive to azole-resistant isolates. In total, 14 SNP loci were detected in this clinical study, which included 3 (i.e., *ERG11*-395, *ERG11*-461, and *ERG11*-769) previously confirmed by gene editing to confer azole resistance. The results of global antifungal monitoring showed that azole resistance of *C. tropicalis* is mainly due to the A395T substitution of *ERG11* and overexpression of Erg11 (34, 35). This drug-resistant mutation is concentrated in Thailand (36). A study conducted in China indicated that A395T and C461T were the most commonly reported non-homologous mutations to *ERG11* related to azole resistance. The independent functions of these two sites were also investigated. The *ERG11* A395T substitution, but not the C461T mutation, was associated with azole resistance. However, these mutations often appear in conjunction. To date, there has been no report of an isolated C461T mutation (37). In this study and in a previous research, the A395T, C461T, and T769C substitutions of *ERG11* were found only in azole-resistant strains. However, in contrast to previous studies, the C461T substitution was independently present in two azole-resistant isolates (i.e., ECIFIG1411 and EC21010386), suggesting the possibility of other resistance mechanisms in these two strains. Isolate ECIFIG1411, but not EC21010386, carried the *ERG11*-T769C mutation, which has been associated with azole resistance. However, EC21010386 had the *UPC2*-T1029C mutation, which was found in one isolate that was resistant to azoles and another isolate that was susceptible to fluconazole but intermediate to voriconazole. These results indicate that the *UPC2*-T1029C mutation is probably linked to azole resistance. Also, some azole-resistant isolates did not carry SNPs at the *ERG11*-395, *ERG11*-461, and *ERG11*-769 loci but rather other azole-associated mutations. The mutation at locus *ERG11*-433 was unique to the azole-resistant isolates ECIFIG267 and ECIFIG298 and was not carried by any other azole-sensitive isolate, suggesting that this locus is important for azole resistance. In this study, mutations at position 1949 of the *FKS1* gene were identified in two isolates that exhibited resistance to anidulafungin and micafungin, consistent with previous findings that *FKS1* mutations led to echinocandin resistance (36). Additionally, a mutation at position 1960 of the *FKS1* gene was detected in one isolate with intermediate to caspofungin, a mutation that has been associated with resistance in other studies (11). Notably, four isolates with mutations at position 1949 of the *FKS1* gene were not resistant to echinocandins, suggesting that this specific mutation might not drastically alter the conformation of enzymes to confer full resistance but rather might cause only partial structural changes, leading to decreased susceptibility but not complete resistance. Also, echinocandin resistance may not solely depend on *FKS1* mutations but could involve other resistance mechanisms (38). Moreover, four isolates exhibited reduced susceptibility to echinocandins but had no *FKS1* mutations, indicating the possibility of mutations at other sites of the *FKS1* gene, cellular stress response, or the upregulation of multi-drug transporters contributing to resistance (39).

MALDI-TOF MS provides highly automated processes and can be applied for multiplex assays, reducing time and cost while increasing sample throughput. The established MALDI-TOF MS-based detection panel is both practical and feasible for rapid and large-scale scanning of mutations of *C. tropicalis* related to drug resistance. Epidemiological monitoring of resistance to antifungal agents is especially important in regions where echinocandin-resistant strains have become increasingly prevalent. However, the study has some limitations. During the performance validation of the panel, it was discovered that due to base mismatches, the panel may incorrectly identify non-mutation as heterozygous mutation and vice versa, which could result in the misinterpretation of results. Given the diverse mechanisms of drug resistance in *C. tropicalis*, this panel currently includes only the most significant mutations, potentially overlooking less common or emerging resistance mechanisms. Additionally, the panel is designed to detect mutations from pure cultures of *C. tropicalis*, which may limit direct application in clinical settings. Further efforts will be required to adapt this method for detecting SNPs directly from clinical specimens, such as blood or tissue samples, to enhance its clinical utility.

## MATERIALS AND METHODS

### Isolates

*C. tropicalis* isolates were collected from patients with invasive fungal infections in the Eastern China Invasive Fungi Infection Group (ECIFIG). Patients with invasive fungal disease were identified in accordance with the definitions of definitive diagnoses of invasive fungal diseases by the European Organization for Research and Treatment of Cancer and the Mycoses Study Group Education and Research Consortium (40). Each isolate was identified by MALDI-TOF MS (Zybio, Inc., Chongqing, China). Antifungal susceptibility of the *C. tropicalis* isolates was determined using the Sensititre YeastOne panel (Thermo Fisher Scientific, Waltham, MA, USA), which has been verified against the M27-Ed4, “Reference Method for Broth Dilution Antifungal Susceptibility Testing of Yeasts; Fourth Informational Supplement,” and the European Committee on Antimicrobial Susceptibility Testing of yeasts (v.7.3.1 valid from 15 January 2017 to 22 April 2020) (41). The susceptibilities of the *C. tropicalis* isolates to antifungal agents are shown in Table S1. To evaluate the repeatability of the method, 10 isolates of *C. tropicalis* from different years and with varying antifungal resistance phenotypes were selected (Table S1), ensuring a balanced representation of resistance profiles. Additionally, 20 isolates were chosen to assess consistency, also derived from diverse years and resistance phenotypes, maximizing the detection of the included SNP sites. To evaluate the clinical performance of the method, all *C. tropicalis* isolates collected between 2017 and 2021 by the ECIFIG were included.

### Target gene selection for antifungal susceptibility

The following seven genes of *C. tropicalis* isolate MYA-3404 associated with antifungal resistance were selected from the National Center for Biotechnology Information database for detection of SNPs: *ERG11* (gene accession number M23673), *FKS1* (gene accession number EU676168.2), *TAC1* (gene accession number XM_002550963.1), *MRR1* (gene accession number XM_002547926.1), *ERG3* (gene accession number XM_002550136), *UPC2* (gene accession number NW_003020056.1), and *MDR1* (gene accession number XM_002548069). In total, 36 SNPs of these genes were selected, which included 9 SNPs previously associated with antifungal resistance through gene editing and 27 others related to antifungal resistance of *C. tropicalis* (Table 3).

**TABLE 3:** List of 36 SNPs of seven *C. tropicalis* genes associated with resistance to azoles and echinocandins

Genes	SNPs	Amino acid substitutions	Phenotype	Confirmed	References
				Level	
FKS1	T1960C	S654P	Echinocandin resistant	Epidemiology	(42, 43)
	T1958G	L653W	Echinocandin resistant	Epidemiology	(11, 44)
	T1949C	F650S	Echinocandin resistant	Epidemiology	(45)
*ERG3*	C774T	S258F	Azole resistant	Gene editing	(46)
	C773T	S258F	Azole resistant	Epidemiology	(47)
*MRR1*	G1939T	A647S	Azole resistant	Epidemiology	(48)
*TAC1*	A491T	N164I	Azole resistant	Epidemiology	(48)
*MDR1*	T227C	V76A	Azole resistant	Epidemiology	(36)
*ERG11*	A395T	Y132F	Azole resistant	Gene editing	(37)
	C461T	S154F	Azole resistant	gene editing	(49, 50)
	T433C	F145L	Azole resistant	Epidemiology	(51)
	C1286T	S429F	Azole resistant	Epidemiology	(51, 52)
	A427C	K143X	Azole resistant	Epidemiology	(53)
	A428G	K143R	Azole resistant	Gene editing	(49, 50)
	T1334A	D445V	Azole resistant	Epidemiology	(37)
	A1172-	∆126aa	Azole resistant	Epidemiology	(46, 47)
	∆132 bp (NA 824–955)	∆44aa (AA276-319)	Azole resistant	Gene editing	(46)
	∆132 bp (NA 824–955)	D275V	Azole resistant	Epidemiology	(54)
	G1391A	G464D	Azole resistant	Epidemiology	(54)
	T374C	V125A	Azole resistant	Gene editing	(54)
	T769C	Y257H	Azole resistant	Gene editing	(54)
	G1390A	G464S	Azole resistant	Gene editing	(55)
	C997A	L333I	Azole resistant	Epidemiology	This study
	G1032C/T	K344N	Azole resistant	Epidemiology	(55)
	G1084A	V362M	Azole resistant	Epidemiology	(37, 54)
	T1086G	V362M	Azole resistant	Epidemiology	(46)
*UPC2*	A1020T	Q340H	Azole resistant	Epidemiology	(54)
	A1141T	T381S	Azole resistant	Epidemiology	(54)
	T1712A	F571Y	Azole suscetible dose dependent	Epidemiology	(48)
	G787A	A263T	Azole resistant	Epidemiology	(54)
	G751A	A251T	Azole resistant	Epidemiology	(54)
	A866T	Q289L	Azole resistant	Epidemiology	(48)
	G889T	A297S	Azole resistant	Epidemiology	(54)
	G1029C/T	L343F	Azole resistant	Epidemiology	(48)
	C1178T	T393I	Azole resistant	Gene editing	(54, 56)
	C560T	S187L	Azole resistant	Epidemiology	(48)

### Design of PCR amplification primers and mass probes

Multiplex PCR primers and mass probes (Table S2) were designed using BatchPrimer3 v.1.0 software (http://wheat.pw.usda.gov/demos/BatchPrimer3/). Also, 15 sets of PCR primers were designed to amplify the target sites for multiplex PCR. The lengths of the primers ranged from 16 to 25 bp, with a 10-bp fixed sequence (ACGTTGGATG) added to the 5′ end of each primer. The lengths of the mass probes were between 14 and 27 bp. The molecular weight was designed to be 4–9 kDa with a minimal difference of 16 Da among the probes.

### PCR-MALDI-TOF MS and data analysis

The operation procedure mainly included DNA extraction, multiplex PCR amplification, mass probe extension (MPE), MALDI–TOF MS data acquisition, and QuanSNP analysis. The study protocol was largely similar to a previously reported method but with minor modifications (24). Total DNA was extracted from all pure cultures of *C. tropicalis* using the phenol-chloroform extraction method, following standard protocols with minor modifications. Briefly, colonies were scraped from Sabouraud dextrose agar, re-suspended in 500 µL of phosphate-buffered saline, and pelleted by centrifugation. The pellet was re-suspended in 500 µL of lysis buffer (50-mM Tris-HCl, pH 8.0, 50-mM ethylenediaminetetraacetic acid, and 1% sodium dodecyl sulfate), and 0.2-µm glass beads were added. Cells were lysed using the FastPrep-24 5G Bead Beating Grinder and Lysis System (MP Biomedicals, Santa Ana, CA, USA). The lysate was extracted with phenol:chloroform: alcohol (25:24:1, vol/vol) and centrifuged. The aqueous phase was transferred and re-extracted with chloroform. DNA was precipitated with absolute ethanol, washed with 75% ethanol, air-dried, and re-suspended in 50 µL of TE buffer (10 mM Tris-Cl, pH 8.0, 1-mM EDTA). DNA concentration and purity were measured using a Quawell Q5000 Micro-Volume UV-Vis Spectrophotometer (Quawell Technology, Sunnyvale, CA, USA). The DNA was considered sufficiently pure at A260/280 = 1.6–2.0 and A260/230 >1. Nucleic acid-free water was used as a blank control. The regions surrounding the target genes were amplified by multiplex PCR. Each reaction volume included 2 µL of DNA (5–10 ng/mL), 2 µL of buffer (Intelligene Biosystems Co., Ltd., Qingdao, China), and 1 µL of primers. The amplification protocol included an initial denaturation step at 95°C for 15 min followed by 30 cycles at 95°C for 15 s, 59°C for 30 s, 72°C for 30 s, and 60°C for 10 min, and then cooled to 4°C. To eliminate free deoxynucleotide triphosphates, the PCR products were digested with 2 mL of shrimp alkaline phosphatase at 37°C for 40 min and 85°C for 5 min, and then cooled to 4°C. Finally, the digested PCR products were mixed with 4 mL of MPE (1 µL of enzyme-linked dideoxynucleotide triphosphate, 1.4 µL of MPE buffer, 0.6 µL of enzyme, and 1 µL of MPE primers) and heated to 95°C for 30 s, followed by five cycles at 95°C for 5 s and 52°C 5 s, and 40 cycles at 80°C for 5 s and 72°C for 3 min, and then cooled to 4°C. After salt purification, the supernatant was purified with a mixture of 3-hydroxypyridine-2-carboxylic acid (1:1). Then, 1 µL of the mixture was spotted on the target plate. After crystallization, the samples were tested. The MALDI-TOF MS data were acquired by QuanNUA (Intelligene Biosystems Co., Ltd.) and analyzed with a QuanTOF I system (Intelligene Biosystems Co., Ltd.). The system can distinguish different MPE primers and molecular weights after extension of MPE primers and present the peak positions of each primer and extended base on the spectrogram.

### Method establishment and optimization

A MALDI-TOF MS-based method was established for high-throughput detection of mutant loci of *C. tropicalis* associated with resistance to echinocandins and azoles. In total, 36 SNPs of seven genes associated with antifungal resistance were selected (Table 3). Optimal amplification primers and mass probes were designed for these loci. To avoid dimer formation, forward or reverse complementary mass probes were used for different loci in the W1 and W2 reactions, which encompassed 18 and 19 mutated loci, respectively. Two different mass probes for the ERG11-428 locus were used, with each reaction including one of the probes, due to the presence of base mutations in some DNA samples. All multiplex-PCR primer sequences, mass probes, and single-base extensions are shown in Table S2. After optimization, the final concentration of the mass probe most suitable for single base extension was determined (Table S2).

### Repeatability assessment

To evaluate the reproducibility of the assay, inter-assay variability was determined on two different experimental days. Each experimental batch comprised 10 *C*. *tropicalis* isolates, and the assay procedures were independently repeated for each batch. The inter-assay variabilities were then compared to assess the consistency of measurements across different experimental conditions. Intra-assay variability analysis was performed to evaluate the precision and reliability of the assay within the same experimental batch. Three technical replicates were used for 36 loci of the 10 *C*. *tropicalis* isolates within a single test batch.

### Evaluation of concordance

Sanger sequencing was used as the gold standard method to sequence seven target genes from 20 *C*. *tropicalis* isolates for validation. Genomic DNA was extracted from pure cultures using the phenol-chloroform extraction method (as previously described). The primers used for Sanger sequencing are listed in Table S3. The Applied Biosystems T100 Thermal Cycler (Bio-Rad Laboratories, Hercules, CA, USA) was used for conventional PCR setup, and the cycling conditions were one cycle at 95°C for 10  min followed by 40 cycles at 95°C for 30 s, 55°C for 30 s, 72°C for 45 s, and a final extension step at 72°C for 10 min. Subsequently, the same primers were selected for sequencing. Sanger sequencing was outsourced to Sangon Biotech Co., Ltd. (Shanghai, China).

### Statistical analysis

Statistical analysis was conducted with Excel 2021 (Microsoft, Redmond, WA, USA). Sample detection results were obtained from MALDI–TOF MS in Excel format (Intelligene Biosystems, IntelliBio), and then descriptive statistical analysis was conducted.

## Supplementary Material

Reviewer comments

## SUPPLEMENTAL MATERIAL

Tables S1 to S3 spectrum.00764-24-s0001.docx This supplementary material provides a description of the MICs, primer sequences, and mass probes used for the isolates in this study.

OPEN PEER REVIEW reviewer-comments.pdf An accounting of the reviewer comments and feedback.

ASM does not own the copyrights to Supplemental Material that may be linked to, or accessed through, an article. The authors have granted ASM a non-exclusive, world-wide license to publish the Supplemental Material files. Please contact the corresponding author directly for reuse.

## References

[1] Pappas PG, Lionakis MS, Arendrup MC, Ostrosky-Zeichner L, Kullberg BJ. 2018. Invasive candidasis. Nat Rev Dis Primers 4:18026. doi:10.1038/nrdp.2018.26 29749387

[2] Kullberg BJ, Arendrup MC. 2015. Invasive candidasis. N Engl J Med 373:1445–1456. doi:10.1056/NEJMra1315399 26444731

[3] Lass-Flörl C, Kanj SS, Govender NP, Thompson GR III, Ostrosky- Zeichner L, Govrins MA. 2024. Invasive candidasis. Nat Rev Dis Primers 10:20. doi:10.1038/s41572-024-00503-3 38514673

[4] Silva S, Negri M, Henriques M, Oliveira R, Williams DW, Azeredo J. 2011. Adherence and biofilm formation of non-Candida albicans Candida species. Trends Microbiol 19:241–247. doi:10.1016/j.tim.2011.02.003 21411325

[5] Lima R, Ribeiro FC, Colombo AL, de Almeida JN Jr. 2022. The emerging threat antifungal-resistant Candida tropicalis in humans, animals, and environment. Front Fungal Biol 3:957021. doi:10.3389/ffunb.2022.957021 37746212 PMC10512401

[6] Wang Y, Fan X, Wang H, Kudinha T, Mei Y-N, Ni F, Pan Y-H, Gao L-M, Xu H, Kong H-S, Yang Q, Wang W-P, Xi H-Y, Luo Y-P, Ye L-Y, Xiao M, China Hospital Invasive Fungal Surveillance Net (CHIF-NET) Study Group. 2021. Continual decline in azole susceptibility rates in Candida tropicalis over a 9-year period in China. Front Microbiol 12:702839. doi:10.3389/fmicb.2021.702839 34305872 PMC8299486

[7] Chakrabarti A, Sood P, Rudramurthy SM, Chen S, Kaur H, Capoor M, Chhina D, Rao R, Eshwara VK, Xess I, et al.. 2015. Incidence, characteristics and outcome of ICU-acquired candidemia in India. Intensive Care Med 41:285–295. doi:10.1007/s00134-014-3603-2 25510301

[8] Arastehfar A, Hilmioğlu-Polat S, Daneshnia F, Hafez A, Salehi M, Polat F, Yaşar M, Arslan N, Hoşbul T, Ünal N, Metin DY, Gürcan Ş, Birinci A, Koç AN, Pan W, Ilkit M, Perlin DS, Lass-Flörl C. 2020. Recent increase in the prevalence of fluconazole-non-susceptible Candida tropicalis blood isolates in Turkey: clinical implication of azole-non-susceptible and fluconazole tolerant phenotypes and genotyping. Front Microbiol 11:587278. doi:10.3389/fmicb.2020.587278 33123116 PMC7573116

[9] Megri Y, Arastehfar A, Boekhout T, Daneshnia F, Hörtnagl C, Sartori B, Hafez A, Pan W, Lass-Flörl C, Hamrioui B. 2020. Candida tropicalis is the most prevalent yeast species causing candidemia in Algeria: the urgent need for antifungal stewardship and infection control measures. Antimicrob Resist Infect Control 9:50. doi:10.1186/s13756-020-00710-z 32264966 PMC7140370

[10] Garcia-Effron G, Kontoyiannis DP, Lewis RE, Perlin DS. 2008. Caspofungin-resistant Candida tropicalis strains causing breakthrough fungemia in patients at high risk for hematologic malignancies. Antimicrob Agents Chemother 52:4181–4183. doi:10.1128/AAC.00802-08 18794386 PMC2573122

[11] Sfeir MM, Jiménez-Ortigosa C, Gamaletsou MN, Schuetz AN, Soave R, Van Besien K, Small CB, Perlin DS, Walsh TJ. 2020. Breakthrough bloodstream infections caused by echinocandin-resistant Candida tropicalis: an emerging threat to immunocompromised patients with hematological malignancies. J Fungi (Basel) 6:20. doi:10.3390/jof6010020 32024039 PMC7151208

[12] Chen PY, Chuang YC, Wu UI, Sun HY, Wang JT, Sheng WH, Lo HJ, Wang HY, Chen YC, Chang SC. 2019. Clonality of fluconazole-nonsusceptible Candida tropicalis in bloodstream infections, Taiwan, 2011-2017. Emerg Infect Dis 25:1660–1667. doi:10.3201/eid2509.190520 PMC6711239 31441426

[13] Bilal H, Shafiq M, Hou B, Islam R, Khan MN, Khan RU, Zeng Y. 2022. Distribution and antifungal susceptibility pattern of Candida species from mainland China: a systematic analysis. Virulence 13:1573–1589. doi:10.1080/21505594.2022.2123325 36120738 PMC9487756

[14] Shafiei M, Peyton L, Hashemzadeh M, Foroumadi A. 2020. History of the development of antifungal azoles: a review on structures, SAR, and mechanism of action. Bioorg Chem 104:104240. doi:10.1016/j.bioorg.2020.104240 32906036

[15] Szymański M, Chmielewska S, Czyżewska U, Malinowska M, Tylicki A. 2022. Echinocandins - structure, mechanism of action and use in antifungal therapy. J Enzyme Inhib Med Chem 37:876–894. doi:10.1080/14756366.2022.2050224 35296203 PMC8933026

[16] Lee Y, Puumala E, Robbins N, Cowen LE. 2021. Antifungal drug resistance: molecular mechanisms in Candida albicans and beyond. Chem Rev 121:3390–3411. doi:10.1021/acs.chemrev.0c00199 32441527 PMC8519031

[17] Fan X, Dai RC, Zhang S, Geng YY, Kang M, Guo DW, Mei YN, Pan YH, Sun ZY, Xu YC, Gong J, Xiao M. 2023. Tandem gene duplications contributed to high-level azole resistance in a rapidly expanding Candida tropicalis population. Nat Commun 14:8369. doi:10.1038/s41467-023-43380-2 38102133 PMC10724272

[18] CLSI. 2017. Reference method for broth dilution antifungal susceptibility testing of yeats. 4th ed. Wayne, PA: Clinical and Laboratory Standards Institute.

[19] EUCAST. 2023. Definitive document E.Def 7.4. method for the determination of broth dilution minimum inhibitory concentrations of antifungal agents for yeasts. Available from: http://www.eucast.org 10.1111/j.1469-0691.2012.03880.x 22563750

[20] Kwok PY. 1998. Genotyping by mass spectrometry takes flight. Nat Biotechnol 16:1314–1315. doi:10.1038/4276 9853606

[21] Sjöholm MIL, Dillner J, Carlson J. 2008. Multiplex detection of human herpesviruses from archival specimens by using matrix-assisted laser desorption ionization-time of flight mass spectrometry. J Clin Microbiol 46:540–545. doi:10.1128/JCM.01565-07 18094141 PMC2238100

[22] Peng J, Gao L, Guo J, Wang T, Wang L, Yao Q, Zhu H, Jin Q. 2013. Type-specific detection of 30 oncogenic human papillomaviruses by genotyping both E6 and L1 genes. J Clin Microbiol 51:402–408. doi:10.1128/JCM.01170-12 23152557 PMC3553860

[23] Syrmis MW, Moser RJ, Whiley DM, Vaska V, Coombs GW, Nissen MD, Sloots TP, Nimmo GR. 2011. Comparison of a multiplexed MassARRAY system with real-time allele-specific PCR technology for genotyping of Staphylococcus aureus. Clin Microbiol Infect 17:1804–1810. doi:10.1111/j.1469-0691.2011.03521.x 21595795

[24] Zhao F, Zhang J, Wang X, Liu L, Gong J, Zhai Z, He L, Meng F, Xiao D. 2021. A multisite SNP genotyping and macrolide susceptibility gene method for Mycoplasma pneumoniae based on MALDI-TOF MS. i Sci 24:102447. doi:10.1016/j.isci.2021.102447 PMC8105657 33997713

[25] Berkow EL, Lockhart SR, Ostrosky-Zeichner L. 2020. Antifungal susceptibility testing: current approaches. Clin Microbiol Rev 33:e00069-19. doi:10.1128/CMR.00069-19 32349998 PMC7194854

[26] van der Linden JWM, Snelders E, Arends JP, Daenen SM, Melchers WJG, Verweij PE. 2010. Rapid diagnosis of aspergillosis by direct PCR using tissue specimens. J Clin Microbiol 48:1478–1480. doi:10.1128/JCM.02221-09 20107096 PMC2849623

[27] Perlin DS, Wiederhold NP. 2017. Culture-independent molecular methods for detection of antifungal resistance mechanisms and fungal identification. J Infect Dis 216:S458–S465. doi:10.1093/infdis/jix121 28911041

[28] Zhu Y, Hager KM, Manjari SR, Banavali NK, Chaturvedi V, Chaturvedi S. 2023. Development and validation of TaqMan chemistry probe-based rapid assay for the detection of echinocandin-resistance in Candida auris. J Clin Microbiol 61:e0176722. doi:10.1128/jcm.01767-22 36975998 PMC10117040

[29] Després PC, Shapiro RS, Cuomo CA. 2024. New approaches to tackle a rising problem: large-scale methods to study antifungal resistance. PLoS Pathog 20:e1012478. doi:10.1371/journal.ppat.1012478 39236046 PMC11376582

[30] Pristov KE, Ghannoum MA. 2019. Resistance of Candida to azoles and echinocandins worldwide. Clin Microbiol Infect 25:792–798. doi:10.1016/j.cmi.2019.03.028 30965100

[31] Lipworth S, Jajou R, de Neeling A, Bradley P, van der Hoek W, Maphalala G, Bonnet M, Sanchez-Padilla E, Diel R, Niemann S, Iqbal Z, Smith G, Peto T, Crook D, Walker T, van Soolingen D. 2019. SNP-IT tool for identifying subspecies and associated lineages of Mycobacterium tuberculosis complex. Emerg Infect Dis 25:482–488. doi:10.3201/eid2503.180894 30789126 PMC6390766

[32] Rahi P, Prakash O, Shouche YS. 2016. Matrix-assisted laser desorption/ionization time-of-flight mass-spectrometry (MALDI-TOF MS) based microbial identifications: challenges and scopes for microbial ecologists. Front Microbiol 7:1359. doi:10.3389/fmicb.2016.01359 27625644 PMC5003876

[33] Kim S, Misra A. 2007. SNP genotyping: technologies and biomedical applications. Annu Rev Biomed Eng 9:289–320. doi:10.1146/annurev.bioeng.9.060906.152037 17391067

[34] Tseng KY, Liao YC, Chen FC, Chen FJ, Lo HJ. 2022. A predominant genotype of azole-resistant Candida tropicalis clinical strains. Lancet Microbe 3:e646. doi:10.1016/S2666-5247(22)00179-3 35752199

[35] Zhou ZL, Tseng KY, Chen YZ, Tsai DJ, Wu CJ, Chen YC, Peng HL, Yang YL, Hsieh LY, Chen CH, Hsu CH, Wang LS, Cheng MF, Hsu GJ, Kao CC, Hu BS, Lee YT, Liu JW, Liu KS, Miu WC, Yang HM, Yeh YC, Lo HJ. 2022. Genetic relatedness among azole-resistant Candida tropicalis clinical strains in Taiwan from 2014 to 2018. Int J Antimicrob Agents 59:106592. doi:10.1016/j.ijantimicag.2022.106592 35460852

[36] Castanheira M, Deshpande LM, Messer SA, Rhomberg PR, Pfaller MA. 2020. Analysis of global antifungal surveillance results reveals predominance of Candida parapsilosis and Candida tropicalis and country-specific isolate dissemination. Int J Antimicrob Agents 55:105799. doi:10.1016/j.ijantimicag.2019.09.003 31520783

[37] Fan X, Xiao M, Zhang D, Huang J-J, Wang H, Hou X, Zhang L, Kong F, Chen SC-A, Tong Z-H, Xu Y-C. 2019. Molecular mechanisms of azole resistance in Candida tropicalis isolates causing invasive candidiasis in China. Clin Microbiol Infect 25:885–891. doi:10.1016/j.cmi.2018.11.007 30472420

[38] Perlin DS, Rautemaa-Richardson R, Alastruey-Izquierdo A. 2017. The global problem of antifungal resistance: prevalence, mechanisms, and management. Lancet Infect Dis 17:e383–e392. doi:10.1016/S1473-3099(17)30316-X 28774698

[39] Cowen LE, Sanglard D, Howard SJ, Rogers PD, Perlin DS. 2014. Mechanisms of antifungal drug resistance. Cold Spring Harb Perspect Med 5:a019752. doi:10.1101/cshperspect.a019752 25384768 PMC4484955

[40] Donnelly JP, Chen SC, Kauffman CA, Steinbach WJ, Baddley JW, Verweij PE, Clancy CJ, Wingard JR, Lockhart SR, Groll AH, et al.. 2020. Revision and update of the consensus definitions of invasive fungal disease from the European organization for research and treatment of cancer and the mycoses study group education and research consortium. Clin Infect Dis 71:1367–1376. doi:10.1093/cid/ciz1008 31802125 PMC7486838

[41] Córdoba S, Abiega C, Agorio I, Amigot S, Ardizzoli K, Giusiano G, Guelfand L, López Moral L, Maldonado I, Pineda G, Garcia-Effron G, Subcomisión de Micología Clínica. 2022. Usefulness of the Sensititre YeastOne panel to detect Candida species resistant to antifungal drugs. Rev Argent Microbiol 54:9–14. doi:10.1016/j.ram.2021.02.002 33875292

[42] Garcia-Effron G, Chua DJ, Tomada JR, DiPersio J, Perlin DS, Ghannoum M, Bonilla H. 2010. Novel FKS mutations associated with echinocandin resistance in Candida species. Antimicrob Agents Chemother 54:2225–2227. doi:10.1128/AAC.00998-09 20145084 PMC2863628

[43] Pfaller MA, Diekema DJ, Turnidge JD, Castanheira M, Jones RN. 2019. Twenty years of the SENTRY antifungal surveillance program: results for Candida species from 1997-2016. Open Forum Infect Dis 6:S79–S94. doi:10.1093/ofid/ofy358 30895218 PMC6419901

[44] Desnos-Ollivier M, Bretagne S, Raoux D, Hoinard D, Dromer F, Dannaoui E, European Committee on Antibiotic Susceptibility Testing. 2008. Mutations in the fks1 gene in Candida albicans, C. tropicalis, and C. krusei correlate with elevated caspofungin MICs uncovered in AM3 medium using the method of the European Committee on Antibiotic Susceptibility Testing. Antimicrob Agents Chemother 52:3092–3098. doi:10.1128/AAC.00088-08 18591282 PMC2533459

[45] Xiao M, Fan X, Hou X, Chen SC, Wang H, Kong F, Sun ZY, Chu YZ, Xu YC. 2018. Clinical characteristics of the first cases of invasive candidiasis in China due to pan-echinocandin-resistant Candida tropicalis and Candida glabrata isolates with delineation of their resistance mechanisms. Infect Drug Resist 11:155–161. doi:10.2147/IDR.S152785 29416360 PMC5790075

[46] Forastiero A, Mesa-Arango AC, Alastruey-Izquierdo A, Alcazar-Fuoli L, Bernal-Martinez L, Pelaez T, Lopez JF, Grimalt JO, Gomez-Lopez A, Cuesta I, Zaragoza O, Mellado E. 2013. Candida tropicalis antifungal cross-resistance is related to different azole target (Erg11p) modifications. Antimicrob Agents Chemother 57:4769–4781. doi:10.1128/AAC.00477-13 23877676 PMC3811422

[47] Eddouzi J, Parker JE, Vale-Silva LA, Coste A, Ischer F, Kelly S, Manai M, Sanglard D. 2013. Molecular mechanisms of drug resistance in clinical Candida species isolated from Tunisian hospitals. Antimicrob Agents Chemother 57:3182–3193. doi:10.1128/AAC.00555-13 23629718 PMC3697321

[48] Arastehfar A, Daneshnia F, Hafez A, Khodavaisy S, Najafzadeh MJ, Charsizadeh A, Zarrinfar H, Salehi M, Shahrabadi ZZ, Sasani E, Zomorodian K, Pan W, Hagen F, Ilkit M, Kostrzewa M, Boekhout T. 2020. Antifungal susceptibility, genotyping, resistance mechanism, and clinical profile of Candida tropicalis blood isolates. Med Mycol 58:766–773. doi:10.1093/mmy/myz124 31828316 PMC7398758

[49] Jiang C, Dong D, Yu B, Cai G, Wang X, Ji Y, Peng Y. 2013. Mechanisms of azole resistance in 52 clinical isolates of Candida tropicalis in China. J Antimicrob Chemother 68:778–785. doi:10.1093/jac/dks481 23221625

[50] You L, Qian W, Yang Q, Mao L, Zhu L, Huang X, Jin J, Meng H. 2017. ERG11 gene mutations and MDR1 upregulation confer pan-azole resistance in Candida tropicalis causing disseminated candidiasis in an acute lymphoblastic leukemia patient on posaconazole prophylaxis. Antimicrob Agents Chemother 61:e02496-16. doi:10.1128/AAC.02496-16 28507109 PMC5487663

[51] Xisto MIDS, Caramalho RDF, Rocha DAS, Ferreira-Pereira A, Sartori B, Barreto-Bergter E, Junqueira ML, Lass-Flörl C, Lackner M. 2017. Pan-azole-resistant Candida tropicalis carrying homozygous erg11 mutations at position K143R: a new emerging superbug? J Antimicrob Chemother 72:988–992. doi:10.1093/jac/dkw558 28065893

[52] Derkacz D, Bernat P, Krasowska A. 2022. K143R amino acid substitution in 14-α-demethylase (Erg11p) changes plasma membrane and cell wall structure of Candida albicans. Int J Mol Sci 23:1631. doi:10.3390/ijms23031631 35163552 PMC8836035

[53] Teo JQ-M, Lee SJ-Y, Tan A-L, Lim RS-M, Cai Y, Lim T-P, Kwa AL-H. 2019. Molecular mechanisms of azole resistance in Candida bloodstream isolates. BMC Infect Dis 19:63. doi:10.1186/s12879-019-3672-5 30654757 PMC6337757

[54] Choi MJ, Won EJ, Shin JH, Kim SH, Lee WG, Kim MN, Lee K, Shin MG, Suh SP, Ryang DW, Im YJ. 2016. Resistance mechanisms and clinical features of fluconazole-nonsusceptible Candida tropicalis isolates compared with fluconazole-less-susceptible isolates. Antimicrob Agents Chemother 60:3653–3661. doi:10.1128/AAC.02652-15 27044550 PMC4879413

[55] Navarro-Rodríguez P, López-Fernández L, Martin-Vicente A, Guarro J, Capilla J. 2020. ERG11 polymorphism in voriconazole-resistant Candida tropicalis: weak role of ERG11 expression, ergosterol content, and membrane permeability. Antimicrob Agents Chemother 65:e00325-20. doi:10.1128/AAC.00325-20 33077654 PMC7927814

[56] Jiang C, Ni Q, Dong D, Zhang L, Li Z, Tian Y, Peng Y. 2016. The role of Candida tropicalis gene in azole-resistant Candida tropicalis. Mycopathologia 181:833–838. doi:10.1007/s11046-016-0050-3 27538831

