# Supplementary material for: A MALDI-TOF MS-based multiple detection panel of drug resistance-associated multiple single-nucleotide polymorphisms in Candida tropicalis
Source: Microbiol Spectr. 2024 Dec 6;13(1):e00764-24. doi: 10.1128/spectrum.00764-24 (PMC11705899; doi:10.1128/spectrum.00764-24)
Supplement: Tables S1 to S3 — This supplementary material provides a description of the MICs, primer sequences, and mass probes used for the isolates in this study. [file spectrum.00764-24-s0001.docx]

**Supplementary Table S1.** Details of antifungal agent susceptibility data.

| Strain | Year of isolation | Application | ANI  (μg/mL) | ANI  susceptibility | CAS  (μg/mL) | CAS  susceptibility | FLU  (μg/mL) | FLU  susceptibility | MICA  (μg/mL) | MICA  susceptibility | VOR  (μg/mL) | VOR  susceptibility |
| --- | --- | --- | --- | --- | --- | --- | --- | --- | --- | --- | --- | --- |
| ECIFIG31 | 2017 | Repeatability evaluation | 0.12 | S | 0.06 | S | 1 | S | 0.03 | S | 0.12 | S |
| ECIFIG343 | 2018 | Repeatability evaluation | 0.12 | S | 0.06 | S | 2 | S | 0.015 | S | 0.12 | S |
| ECIFIG685 | 2018 | Repeatability evaluation | 0.25 | S | 0.25 | S | 128 | R | 0.03 | S | 8 | R |
| ECIFIG687 | 2018 | Repeatability evaluation | 0.25 | S | 0.12 | S | 256 | R | 0.03 | S | 8 | R |
| ECIFIG1112 | 2019 | Repeatability evaluation | 0.12 | S | 0.12 | S | 256 | R | 0.06 | S | 8 | R |
| ECIFIG1448 | 2019 | Repeatability evaluation | 0.12 | S | 0.12 | S | 1 | S | 0.03 | S | 0.06 | S |
| ECIFIG1642 | 2020 | Repeatability evaluation | 1 | R | 0.12 | S | 1 | S | 0.03 | S | 0.06 | S |
| ECIFIG1831 | 2020 | Repeatability evaluation | 0.12 | S | 0.12 | S | 256 | R | 0.03 | S | 8 | R |
| ECIFIG21032 | 2021 | Repeatability evaluation | 1 | R | 0.5 | I | 8 | R | 1 | R | 0.5 | I |
| ECIFIG21340 | 2021 | Repeatability evaluation | 0.25 | S | 0.06 | S | 4 | SDD | 0.06 | S | 0.25 | I |
| ECIFIG27 | 2017 | Coherence assessment | 0.12 | S | 0.12 | S | 4 | SDD | 0.03 | S | 0.25 | I |
| ECIFIG113 | 2017 | Coherence assessment | 0.12 | S | 0.25 | S | 2 | S | 0.03 | S | 0.25 | I |
| ECIFIG158 | 2017 | Coherence assessment | 0.25 | S | 0.12 | S | 128 | R | 0.03 | S | 8 | R |
| ECIFIG159 | 2017 | Coherence assessment | 0.12 | S | 0.12 | S | 32 | R | 0.03 | S | 2 | R |
| ECIFIG189 | 2017 | Coherence assessment | 0.12 | S | 0.06 | S | 4 | SDD | 0.03 | S | 0.25 | I |
| ECIFIG196 | 2017 | Coherence assessment | 0.25 | S | 0.25 | S | 256 | R | 0.03 | S | 8 | R |
| ECIFIG214 | 2017 | Coherence assessment | 0.12 | S | 0.06 | S | 4 | SDD | 0.015 | S | 0.12 | S |
| ECIFIG260 | 2018 | Coherence assessment | 0.25 | S | 0.25 | S | 256 | R | 0.03 | S | 8 | R |
| ECIFIG354 | 2018 | Coherence assessment | 0.12 | S | 0.25 | S | 4 | SDD | 0.03 | S | 0.25 | I |
| ECIFIG358 | 2018 | Coherence assessment | 0.25 | S | 0.25 | S | 256 | R | 0.06 | S | 8 | R |
| ECIFIG385 | 2018 | Coherence assessment | 0.12 | S | 0.12 | S | 4 | SDD | 0.03 | S | 0.25 | I |
| ECIFIG455 | 2018 | Coherence assessment | 0.25 | S | 0.06 | S | 4 | SDD | 0.03 | S | 0.12 | S |
| ECIFIG760 | 2018 | Coherence assessment | 0.25 | S | 0.5 | I | 4 | SDD | 0.03 | S | 0.25 | I |
| ECIFIG818 | 2018 | Coherence assessment | 0.12 | S | 0.25 | S | 4 | SDD | 0.03 | S | 0.5 | I |
| ECIFIG833 | 2018 | Coherence assessment | 0.12 | S | 0.12 | S | 4 | SDD | 0.03 | S | 0.12 | S |
| ECIFIG844 | 2018 | Coherence assessment | 0.12 | S | 0.06 | S | 8 | R | 0.03 | S | 0.5 | I |
| ECIFIG885 | 2019 | Coherence assessment | 0.25 | S | 0.12 | S | 256 | R | 0.06 | S | 8 | R |
| ECIFIG889 | 2019 | Coherence assessment | 0.12 | S | 0.25 | S | 256 | R | 0.03 | S | 8 | R |
| ECIFIG909 | 2019 | Coherence assessment | 0.12 | S | 0.12 | S | 256 | R | 0.015 | S | 8 | R |
| ECIFIG1521 | 2020 | Coherence assessment | 1 | R | 4 | R | 256 | R | 2 | R | 8 | R |
| ECIFIG29 | 2017 | Clinical performance evaulation | 0.12 | S | 0.12 | S | 128 | R | 0.03 | S | 8 | R |
| ECIFIG56 | 2017 | Clinical performance evaulation | 0.5 | I | 0.5 | I | 16 | R | 0.03 | S | 0.5 | I |
| ECIFIG88 | 2017 | Clinical performance evaulation | 0.25 | S | 0.12 | S | 128 | R | 0.03 | S | 8 | R |
| ECIFIG102 | 2017 | Clinical performance evaulation | 0.12 | S | 0.12 | S | 128 | R | 0.03 | S | 8 | R |
| ECIFIG236 | 2018 | Clinical performance evaulation | 0.25 | S | 0.06 | S | 2 | S | 0.03 | S | 0.12 | S |
| ECIFIG239 | 2018 | Clinical performance evaulation | 0.25 | S | 0.06 | S | 128 | R | 0.03 | S | 1 | R |
| ECIFIG267 | 2018 | Clinical performance evaulation | 0.25 | S | 0.25 | S | 8 | R | 0.03 | S | 1 | R |
| ECIFIG288 | 2018 | Clinical performance evaulation | 0.12 | S | 0.12 | S | 1 | S | 0.03 | S | 0.06 | S |
| ECIFIG294 | 2018 | Clinical performance evaulation | 0.25 | S | 0.12 | S | 256 | R | 0.03 | S | 8 | R |
| ECIFIG296 | 2018 | Clinical performance evaulation | 0.12 | S | 0.03 | S | 16 | R | 0.03 | S | 1 | R |
| ECIFIG297 | 2018 | Clinical performance evaulation | 0.12 | S | 0.12 | S | 2 | S | 0.03 | S | 0.12 | S |
| ECIFIG298 | 2018 | Clinical performance evaulation | 0.12 | S | 0.12 | S | 32 | R | 0.015 | S | 1 | R |
| ECIFIG299 | 2018 | Clinical performance evaulation | 0.25 | S | 0.25 | S | 256 | R | 0.03 | S | 8 | R |
| ECIFIG300 | 2018 | Clinical performance evaulation | 0.12 | S | 0.12 | S | 1 | S | 0.03 | S | 0.03 | S |
| ECIFIG301 | 2018 | Clinical performance evaulation | 0.12 | S | 0.12 | S | 256 | R | 0.03 | S | 8 | R |
| ECIFIG303 | 2018 | Clinical performance evaulation | 0.12 | S | 0.12 | S | 256 | R | 0.03 | S | 8 | R |
| ECIFIG312 | 2018 | Clinical performance evaulation | 0.12 | S | 0.06 | S | 256 | R | 0.03 | S | 8 | R |
| ECIFIG313 | 2018 | Clinical performance evaulation | 0.25 | S | 0.12 | S | 16 | R | 0.03 | S | 0.5 | I |
| ECIFIG373 | 2018 | Clinical performance evaulation | 0.25 | S | 0.12 | S | 4 | SDD | 0.03 | S | 0.5 | I |
| ECIFIG377 | 2018 | Clinical performance evaulation | 0.25 | S | 0.12 | S | 256 | R | 0.06 | S | 8 | R |
| ECIFIG381 | 2018 | Clinical performance evaulation | 0.25 | S | 0.25 | S | 256 | R | 0.03 | S | 8 | R |
| ECIFIG395 | 2018 | Clinical performance evaulation | 0.25 | S | 0.25 | S | 4 | SDD | 0.06 | S | 0.25 | I |
| ECIFIG415 | 2018 | Clinical performance evaulation | 0.25 | S | 0.06 | S | 1 | S | 0.03 | S | 0.06 | S |
| ECIFIG509 | 2018 | Clinical performance evaulation | 0.25 | S | 0.12 | S | 128 | R | 0.06 | S | 8 | R |
| ECIFIG534 | 2018 | Clinical performance evaulation | 0.12 | S | 0.06 | S | 4 | SDD | 0.03 | S | 0.25 | I |
| ECIFIG551 | 2018 | Clinical performance evaulation | 0.12 | S | 0.12 | S | 8 | R | 0.015 | S | 1 | R |
| ECIFIG630 | 2018 | Clinical performance evaulation | 0.25 | S | 0.12 | S | 256 | R | 0.03 | S | 8 | R |
| ECIFIG634 | 2018 | Clinical performance evaulation | 1 | R | 2 | R | 2 | S | 1 | R | 0.06 | S |
| ECIFIG682 | 2018 | Clinical performance evaulation | 0.25 | S | 0.5 | I | 16 | R | 0.03 | S | 1 | R |
| ECIFIG717 | 2018 | Clinical performance evaulation | 0.12 | S | 0.12 | S | 256 | R | 0.03 | S | 8 | R |
| ECIFIG729 | 2018 | Clinical performance evaulation | 0.25 | S | 0.25 | S | 128 | R | 0.03 | S | 4 | R |
| ECIFIG733 | 2018 | Clinical performance evaulation | 0.12 | S | 0.06 | S | 128 | R | 0.03 | S | 8 | R |
| ECIFIG794 | 2018 | Clinical performance evaulation | 0.12 | S | 0.12 | S | 128 | R | 0.03 | S | 8 | R |
| ECIFIG801 | 2018 | Clinical performance evaulation | 0.12 | S | 0.06 | S | 256 | R | 0.06 | S | 8 | R |
| ECIFIG804 | 2018 | Clinical performance evaulation | 0.12 | S | 0.12 | S | 4 | SDD | 0.03 | S | 0.25 | I |
| ECIFIG848 | 2018 | Clinical performance evaulation | 0.12 | S | 0.06 | S | 256 | R | 0.03 | S | 8 | R |
| ECIFIG883 | 2019 | Clinical performance evaulation | 0.12 | S | 0.12 | S | 256 | R | 0.03 | S | 8 | R |
| ECIFIG890 | 2019 | Clinical performance evaulation | 0.12 | S | 0.25 | S | 256 | R | 0.03 | S | 8 | R |
| ECIFIG898 | 2019 | Clinical performance evaulation | 0.12 | S | 0.25 | S | 256 | R | 0.03 | S | 8 | R |
| ECIFIG931 | 2019 | Clinical performance evaulation | 0.12 | S | 0.25 | S | 16 | R | 0.015 | S | 1 | R |
| ECIFIG953 | 2019 | Clinical performance evaulation | 0.12 | S | 0.06 | S | 256 | R | 0.06 | S | 8 | R |
| ECIFIG965 | 2019 | Clinical performance evaulation | 0.25 | S | 0.12 | S | 256 | R | 0.06 | S | 8 | R |
| ECIFIG1005 | 2019 | Clinical performance evaulation | 2 | R | 0.5 | I | 0.5 | S | 1 | R | 0.008 | S |
| ECIFIG1012 | 2019 | Clinical performance evaulation | 0.12 | S | 0.12 | S | 2 | S | 0.06 | S | 0.12 | S |
| ECIFIG1060 | 2019 | Clinical performance evaulation | 0.06 | S | 0.06 | S | 2 | S | 0.06 | S | 0.06 | S |
| ECIFIG1130 | 2019 | Clinical performance evaulation | 0.12 | S | 0.12 | S | 256 | R | 0.06 | S | 8 | R |
| ECIFIG1200 | 2019 | Clinical performance evaulation | 0.06 | S | 0.25 | S | 1 | S | 0.03 | S | 0.06 | S |
| ECIFIG1213 | 2019 | Clinical performance evaulation | 0.12 | S | 0.12 | S | 2 | S | 0.06 | S | 0.25 | I |
| ECIFIG1222 | 2019 | Clinical performance evaulation | 0.03 | S | 0.03 | S | 8 | R | 0.03 | S | 0.5 | I |
| ECIFIG1258 | 2019 | Clinical performance evaulation | 0.12 | S | 0.25 | S | 128 | R | 0.06 | S | 8 | R |
| ECIFIG1297 | 2019 | Clinical performance evaulation | 0.12 | S | 0.25 | S | 128 | R | 0.06 | S | 8 | R |
| ECIFIG1306 | 2019 | Clinical performance evaulation | 0.12 | S | 0.25 | S | 4 | SDD | 0.06 | S | 0.25 | I |
| ECIFIG1309 | 2019 | Clinical performance evaulation | 0.12 | S | 0.12 | S | 4 | SDD | 0.03 | S | 0.25 | I |
| ECIFIG1312 | 2019 | Clinical performance evaulation | 0.12 | S | 0.12 | S | 256 | R | 0.06 | S | 8 | R |
| ECIFIG1353 | 2019 | Clinical performance evaulation | 0.12 | S | 0.12 | S | 256 | R | 0.03 | S | 8 | R |
| ECIFIG1371 | 2019 | Clinical performance evaulation | 0.12 | S | 0.25 | S | 4 | SDD | 0.03 | S | 0.5 | I |
| ECIFIG1374 | 2019 | Clinical performance evaulation | 0.12 | S | 0.12 | S | 4 | SDD | 0.06 | S | 0.5 | I |
| ECIFIG1392 | 2019 | Clinical performance evaulation | 0.12 | S | 0.25 | S | 2 | S | 0.06 | S | 0.12 | S |
| ECIFIG1394 | 2019 | Clinical performance evaulation | 0.12 | S | 0.25 | S | 2 | S | 0.06 | S | 0.12 | S |
| ECIFIG1408 | 2019 | Clinical performance evaulation | 0.12 | S | 0.12 | S | 256 | R | 0.03 | S | 8 | R |
| ECIFIG1410 | 2019 | Clinical performance evaulation | 0.12 | S | 0.12 | S | 256 | R | 0.03 | S | 8 | R |
| ECIFIG1411 | 2019 | Clinical performance evaulation | 0.12 | S | 0.12 | S | 32 | R | 0.03 | S | 1 | R |
| ECIFIG1412 | 2019 | Clinical performance evaulation | 0.12 | S | 0.12 | S | 256 | R | 0.03 | S | 8 | R |
| ECIFIG1501 | 2020 | Clinical performance evaulation | 0.12 | S | 0.06 | S | 256 | R | 0.03 | S | 8 | R |
| ECIFIG1558 | 2020 | Clinical performance evaulation | 0.12 | S | 0.06 | S | 1 | S | 0.03 | S | 0.06 | S |
| ECIFIG1596 | 2020 | Clinical performance evaulation | 0.12 | S | 0.06 | S | 2 | S | 0.03 | S | 0.25 | I |
| ECIFIG1625 | 2020 | Clinical performance evaulation | 0.12 | S | 0.06 | S | 1 | S | 0.03 | S | 0.03 | S |
| ECIFIG1680 | 2020 | Clinical performance evaulation | 0.06 | S | 0.06 | S | 2 | S | 0.03 | S | 0.25 | I |
| ECIFIG1692 | 2020 | Clinical performance evaulation | 0.015 | S | 0.12 | S | 2 | S | 0.03 | S | 0.12 | S |
| ECIFIG1694 | 2020 | Clinical performance evaulation | 0.03 | S | 0.06 | S | 1 | S | 0.03 | S | 0.12 | S |
| ECIFIG1699 | 2020 | Clinical performance evaulation | 0.12 | S | 0.06 | S | 4 | SDD | 0.03 | S | 0.25 | I |
| ECIFIG1720 | 2020 | Clinical performance evaulation | 0.03 | S | 0.06 | S | 4 | SDD | 0.03 | S | 0.5 | I |
| ECIFIG1721 | 2020 | Clinical performance evaulation | 0.015 | S | 0.12 | S | 4 | SDD | 0.03 | S | 0.25 | I |
| ECIFIG1766 | 2020 | Clinical performance evaulation | 0.06 | S | 0.06 | S | 2 | S | 0.03 | S | 0.25 | I |
| ECIFIG1769 | 2020 | Clinical performance evaulation | 0.03 | S | 0.06 | S | 4 | SDD | 0.03 | S | 0.25 | I |
| ECIFIG1787 | 2020 | Clinical performance evaulation | 0.25 | S | 0.06 | S | 4 | SDD | 0.03 | S | 0.25 | I |
| ECIFIG1819 | 2020 | Clinical performance evaulation | 0.12 | S | 0.06 | S | 256 | R | 0.06 | S | 8 | R |
| ECIFIG1829 | 2020 | Clinical performance evaulation | 0.12 | S | 0.12 | S | 4 | SDD | 0.03 | S | 0.25 | I |
| ECIFIG1834 | 2020 | Clinical performance evaulation | 0.12 | S | 0.06 | S | 128 | R | 0.06 | S | 8 | R |
| ECIFIG1844 | 2020 | Clinical performance evaulation | 0.25 | S | 0.12 | S | 2 | S | 0.06 | S | 0.25 | I |
| ECIFIG1885 | 2020 | Clinical performance evaulation | 0.12 | S | 0.12 | S | 128 | R | 0.06 | S | 8 | R |
| ECIFIG21021 | 2021 | Clinical performance evaulation | 2 | R | 0.5 | I | 0.5 | S | 1 | R | 0.008 | S |
| ECIFIG21198 | 2021 | Clinical performance evaulation | 0.12 | S | 0.06 | S | 2 | S | 0.03 | S | 0.12 | S |
| ECIFIG21200 | 2021 | Clinical performance evaulation | 0.03 | S | 0.06 | S | 2 | S | 0.03 | S | 0.12 | S |
| ECIFIG21203 | 2021 | Clinical performance evaulation | 0.03 | S | 0.06 | S | 2 | S | 0.03 | S | 0.12 | S |
| ECIFIG21256 | 2021 | Clinical performance evaulation | 0.06 | S | 0.12 | S | 1 | S | 0.03 | S | 0.06 | S |
| ECIFIG21261 | 2021 | Clinical performance evaulation | 0.12 | S | 0.06 | S | 2 | S | 0.03 | S | 0.12 | S |
| ECIFIG21262 | 2021 | Clinical performance evaulation | 0.12 | S | 0.12 | S | 1 | S | 0.03 | S | 0.12 | S |
| ECIFIG21266 | 2021 | Clinical performance evaulation | 0.12 | S | 0.06 | S | 1 | S | 0.03 | S | 0.06 | S |
| ECIFIG21268 | 2021 | Clinical performance evaulation | 0.06 | S | 0.06 | S | 2 | S | 0.03 | S | 0.06 | S |
| ECIFIG21331 | 2021 | Clinical performance evaulation | 0.12 | S | 0.06 | S | 1 | S | 0.03 | S | 0.06 | S |
| ECIFIG21335 | 2021 | Clinical performance evaulation | 0.06 | S | 0.06 | S | 1 | S | 0.03 | S | 0.03 | S |
| ECIFIG21380 | 2021 | Clinical performance evaulation | 0.12 | S | 0.06 | S | 2 | S | 0.03 | S | 0.12 | S |
| ECIFIG21381 | 2021 | Clinical performance evaulation | 0.03 | S | 0.06 | S | 2 | S | 0.03 | S | 0.25 | I |
| ECIFIG21385 | 2021 | Clinical performance evaulation | 0.06 | S | 0.12 | S | 4 | SDD | 0.03 | S | 0.25 | I |
| ECIFIG21388 | 2021 | Clinical performance evaulation | 0.12 | S | 0.06 | S | 2 | S | 0.06 | S | 0.12 | S |
| ECIFIG21413 | 2021 | Clinical performance evaulation | 0.03 | S | 0.06 | S | 2 | S | 0.03 | S | 0.12 | S |
| ECIFIG21438 | 2021 | Clinical performance evaulation | 0.12 | S | 0.06 | S | 2 | S | 0.03 | S | 0.25 | I |
| ECIFIG21010047 | 2021 | Clinical performance evaulation | 0.06 | S | 0.25 | S | 128 | R | 0.03 | S | 1 | R |
| ECIFIG21010155 | 2021 | Clinical performance evaulation | 0.12 | S | 0.06 | S | 128 | R | 0.03 | S | 1 | R |
| ECIFIG21010178 | 2021 | Clinical performance evaulation | 0.015 | S | 0.06 | S | 16 | R | 0.03 | S | 0.5 | I |
| ECIFIG21010208 | 2021 | Clinical performance evaulation | 0.25 | S | 0.12 | S | 128 | R | 0.06 | S | 8 | R |
| ECIFIG21010247 | 2021 | Clinical performance evaulation | 0.06 | S | 0.06 | S | 256 | R | 0.03 | S | 8 | R |
| ECIFIG21010269 | 2021 | Clinical performance evaulation | 1 | R | 0.5 | I | 8 | R | 1 | R | 0.25 | I |
| ECIFIG21010274 | 2021 | Clinical performance evaulation | 1 | R | 0.5 | I | 0.5 | S | 1 | R | 0.008 | S |
| ECIFIG21010315 | 2021 | Clinical performance evaulation | 0.12 | S | 0.25 | S | 128 | R | 0.03 | S | 2 | R |
| ECIFIG21010316 | 2021 | Clinical performance evaulation | 0.25 | S | 0.12 | S | 32 | R | 0.06 | S | 2 | R |
| ECIFIG21010368 | 2021 | Clinical performance evaulation | 0.12 | S | 0.06 | S | 256 | R | 0.03 | S | 8 | R |
| ECIFIG21010386 | 2021 | Clinical performance evaulation | 0.12 | S | 0.06 | S | 128 | R | 0.03 | S | 8 | R |

Abbreviations: ANI, anidulafungin; CAS, caspofungin; FLU, fluconazole; I, intermediate; MICA, micafungin; R, resistant; S, susceptible; SDD, susceptible-dose dependent; VOR, voriconazole

**Supplementary Table S2**. Primer sequences for susceptibility target site amplification and Single-base-extension (SBE) for *C. tropicalis.*

| **Reaction system** | **Target genes** | **Multiple-PCR** | |  | **SBE** | | | | | | | | | |
| --- | --- | --- | --- | --- | --- | --- | --- | --- | --- | --- | --- | --- | --- | --- |
|  |  | **Forward primer sequence** | **Reverse primer sequence** |  | **mass probe（5‘→3’）** | **final concentration of mass probe** | **mass probe direction** | **mass probe mass (Da)** | **Extension call** | **Extended mass**  **(Da)** | **Extension call** | **Extended mass**  **(Da)** | **Extension call** | **Extended mass**  **(Da)** |
| W2 | FKS1-1949T->C | TCCTACTTGTTGTGGTTCCT | CAAGATACATCAAACCCAAGAC |  | gTTGGTTGAATCTTATTTCT | 9.97 | F | 6103 | T | 6445 | C | 6376 |  |  |
| W1 | FKS1-1958T->G |  |  |  | ATCTTATTTCTTCTTGACTT | 9.84 | F | 6014.0 | T | 6356.0 | G | 6327.0 |  |  |
| W1 | FKS1-1960T->C |  |  |  | ATTGGATCTCTTAAAG | 10.04 | R | 4895.2 | TC | 5192.2 | CC | 5208.2 |  |  |
| W1 | ERG3-773C->T | TACATAAACCACATCATAAAT | ATGGGAAAAATAATGGATAGAT |  | TTGTTTGTACTCCTTTTGCAT | 10.35 | F | 6359.2 | C | 6632.2 | T | 6701.2 |  |  |
| W1 | ERG3-774C->T |  |  |  | ACCATCAACTGGATGGAAAGCATG | 12.07 | R | 7394.8 | C | 7707.8 | T | 7691.8 |  |  |
| W2 | MRR1-1939G->T | AGGATGGAGATGACGGAGATG | TGCCAAATCTTTCTTCCCATATT |  | cGGAGCAGATACTTATGCTTTAAAT | 12.59 | F | 7680.0 | G | 7993.0 | T | 8022.0 |  |  |
| W2 | TAC1-491A->T | TCTAAGAGTCATTCAACTGGTGAT | TTCAGGTTCTTCATCTTCCGTAAT |  | cGCTAACTTCTCAAAGTA | 9.03 | F | 5442.6 | A | 5739.6 | T | 5784.6 |  |  |
| W2 | MDR1-227T->C | CGCGTCTAATTCAACAGC | AGCAACAACTCTACCAACACCT |  | aCAATTATTTTACCATCATCA | 10.30 | R | 6323.2 | T | 6620.2 | C | 6636.2 |  |  |
| W1 | ERG11-374T->C | AAATTATCCGATGTTTCTGC | CAAAATAATTCAAAACTTCTTCTCT |  | CTTATACCCATTTGACTACTCCTG | 11.75 | F | 7213.8 | T | 7555.8 | C | 7486.8 |  |  |
| W2 | ERG11-395A->T |  |  |  | gTTTGGTAAAGGTGTTATTT | 10.13 | F | 6208.0 | A | 6505.0 | T | 6550.0 |  |  |
| W1 | ERG11-427A->C |  |  |  | cCCAAACTCTAGATTAATGGAACAA | 12.49 | F | 7627.0 | A | 7924.0 | C | 7900.0 |  |  |
| W1 | ERG11-428.1A->G |  |  |  | cgACTCTAGATTAATGGAACAAA | 11.49 | F | 7064.6 | A | 7361.6 | G | 7377.6 |  |  |
| W2 | ERG11-428.2A->G |  |  |  | cgACTCTAGATTAATGGAACAAA | 11.49 | F | 7064.6 | A | 7361.6 | G | 7377.6 |  |  |
| W2 | ERG11-433T->C |  |  |  | caAAAGCAAATTTAGCAA | 9.12 | R | 5508.6 | T | 5805.6 | C | 5821.6 |  |  |
| W2 | ERG11-461C->T |  |  |  | gCTTTGGAACATAGGTTTTGAAA | 11.58 | R | 7117.6 | C | 7430.6 | T | 7414.6 |  |  |
| W2 | ERG11-769T->C | TCCCAAACTTGCCATTACCTC | TCACCGCTTTCTCTTCTTCTCT |  | CTCTTCTTCTCTTAATTTCCTTCATGT | 13.37 | R | 8083.4 | T | 8380.4 | C | 8396.4 |  |  |
| W1 | ERG11-824A->- | GAAGAATTGACCAACTTGTTGAAAG | CATAGTGACCTTTTGGAATAACAT |  | ATTGATCCAAAGAGAG | 8.36 | F | 4938.2 | A | 5235.2 | DEL | 5280.2 |  |  |
| W2 | ERG11-955T->- |  |  |  | cGTTGTTCAGCCAAATGCAACAAGA | 12.55 | R | 7659.0 | T | 7956.0 | DEL | 7932.0 |  |  |
| W2 | ERG11-997C->A |  |  |  | GTCAATTCTTCGTAAA | 8.26 | R | 4855.2 | C | 5168.2 | A | 5197.2 |  |  |
| W2 | ERG11-1032G->C/T |  |  |  | cGTCAAATCGTTCAAGTCACCACC | 11.80 | R | 7241.8 | G | 7514.8 | C | 7554.8 | T | 7538.8 |
| W1 | ERG11-1084G->A |  |  |  | cACTTGCAAAAATTACCATTG | 10.38 | F | 6373.2 | G | 6686.2 | A | 6670.2 |  |  |
| W2 | ERG11-1086T->G |  |  |  | aTTAATAGTGTTGTT | 7.95 | R | 4612 | T | 4909 | G | 4885 |  |  |
| W2 | ERG11-1172A->- |  |  |  | aGAACCCATTGAGAGTCCCAA | 10.43 | F | 6408.2 | A | 6705.2 | DEL | 6750.2 |  |  |
| W2 | ERG11-1286C->T | ACACCCAGAACATTTCAACCC | TCACCAATACATCTGTGTCTACCAC |  | CCCAAGAAGATGGGAAT | 8.78 | F | 5252.4 | C | 5525.4 | T | 5594.4 |  |  |
| W1 | ERG11-1334T->A |  |  |  | cTCAATTCTGAAGATACTG | 9.51 | F | 5786.8 | T | 6128.8 | A | 6083.8 |  |  |
| W2 | ERG11-1390G->A |  |  |  | CTCCATACTTGCCATTC | 8.52 | F | 5056.4 | G | 5369.4 | A | 5353.4 |  |  |
| W1 | ERG11-1391G->A |  |  |  | gCATCTGTGTCTACCACCA | 9.42 | R | 5723.8 | G | 5996.8 | A | 6065.8 |  |  |
| W1 | UPC2-560C->T | TTCAGGACTTTTAAGTGCAGGGAAT | ATTACCGAGACTTGTG |  | cGATTACTTAAATCCCCTAAACCT | 12.87 | R | 7215.8 | C | 7528.8 | T | 7512.8 |  |  |
| W1 | UPC2-751G->A | GAAATACCCCTACATCCATTCCAAC | GTGCTTGTGATTGTGCTTGCG |  | cTACATCCATTCCAACGAATATCAAC | 9.57 | F | 7827.2 | G | 8140.2 | A | 8124.2 |  |  |
| W1 | UPC2-787G->A |  |  |  | TGTTGCAAATCAGATACTG | 10.67 | R | 5826.8 | G | 6099.8 | A | 6168.8 |  |  |
| W2 | UPC2-866A->T |  |  |  | GCTCCCACCGCAACAGCAAC | 9.82 | F | 6001 | A | 6298 | T | 6343 |  |  |
| W1 | UPC2-889G->T |  |  |  | cCAAGCCCCATCTCAACCACAA | 8.31 | F | 6562.4 | G | 6875.4 | T | 6904.4 |  |  |
| W2 | UPC2-1020A->T | CCACAACAGCAACAGCAACCAC | GTTCATTCCCGGCAAATCCAGC |  | aCATTCAACATCACCTTCA | 9.35 | F | 5675.8 | A | 5972.8 | T | 6017.8 |  |  |
| W1 | UPC2-1029G->C/T |  |  |  | GGCAAATCCAGCTGTGAAGC | 9.26 | R | 6151.0 | G | 6424.0 | C | 6464.0 | T | 6448.0 |
| W1 | UPC2-1141A->T | GCAACAGCAGCTTCAACTTCTT | CGGTGAAATATTAGGTATAGATTTC |  | aAGATGATGTAGGCATAG | 10.13 | R | 5611.6 | A | 5953.6 | T | 5908.6 |  |  |
| W1 | UPC2-1178C->T |  |  |  | gAGGTATAGATTTCGCTGAG | 11.75 | R | 6212.0 | C | 6525.0 | T | 6509.0 |  |  |
| W2 | UPC2-1712T->A | GCCATTAACCGAAGCATCCAGA | AGCTCCTTCACGATCAATACCC |  | AACTGAAATGAACTTATAG | 9.59 | R | 5843.8 | T | 6140.8 | A | 6185.8 |  |  |

**Supplementary Table S3**. Primer sequence list for sanger sequencing of *C. tropicalis* target genes*.*

| **Target genes** | **Forward primer sequence（5‘→3’）** | **Location(bp)** | **Temperature(°C)** | **Reverse primer sequence（5‘→3’）** | **Location(bp)** | **Temperature(°C)** |
| --- | --- | --- | --- | --- | --- | --- |
| FKS1 | TCCGTTGTCGGTTTCTTTAT | 1729 to 1748 | 55.02 | GGCCAAAATCTTGGAGTAGA | 2241 to 2222 | 55.03 |
| ERG3 | CAAATGTCTTTGGAAATCGGT | 493 to 513 | 55.03 | TGCTTCCAAACTTTCTTTTCA | 1080 to 1100 | 54.41 |
| MRR3 | TCAATTCAACTGATCCTTCAGA | 1712 to 1733 | 55.1 | ATAGCCAGGGAAATTCAGAG | 2211 to 2192 | 54.48 |
| TAC1 | ACTGAACGTCCTATGAAGAA | 220 to 239 | 53.74 | AGGAGGATCCACCCATTTTA | 783 to 764 | 55.46 |
| MDR1 | ACAGTTTTGTTGGTAGAATGG | 23 to 43 | 54.3 | ATAAATTGAAGTTCTACCGAAAATG | 522 to 498 | 54.46 |
| ERG11-1 | TCTGACATGGTGTGTGTGTG | 1 to 20 | 62.1 | ATT GAT GCC ATC AAT GGC AG | 659 to 678 | 62.1 |
| ERG11-2 | CACAGTTATAGACCCACAAGGT | 572 to 593 | 50.0 | CAGCGTCACGTCTCCAGTAAT | 1367 to 1387 | 50.0 |
| ERG11-3 | ACTCCTGTTTTTGGTAAAGGTGT | 1009 to 1031 | 49.8-50.5 | GCAATGGCATGTGCATTCTC | 1771 to 1752 | 49.8-50.5 |
| UPC2-1 | AGACGTGTCAAATGTGATGA | 190 to 209 | 55.03 | GCTGTTGCAAAAGGATTTCT | 1109 to 1090 | 55.05 |
| UPC2-2 | TTTGTTGGATCCTCTGATGG | 666 to 685 | 55.02 | GGGGTGTTCAAAGGCTAATA | 1398 to 1379 | 55.01 |
| UPC2-3 | TATTAGCCTTTGAACACCCC | 1379 to 1398 | 55.01 | CAGTAGTGAAAGAACGCAAC | 2068 to 2049 | 54.98 |
